# Environmental enrichment changes rabbits’ behavior, serum hormone level and further affects cecal microbiota

**DOI:** 10.7717/peerj.13068

**Published:** 2022-03-09

**Authors:** Yang Feng, Huimei Fan, Xue Liang, Xiaofeng Wang, Guoyan Gao, Shuangbao Gun

**Affiliations:** 1 College of Animal Science and Technology, Gansu Agricultural University, Lanzhou, Gansu, China; 2 College of Animal Science and Technology, Sichuan Agricultural University, Chengdu, Sichuan, China

**Keywords:** Environmental enrichment, Cecal microbiota, Rabbit husbandry, Rabbit behavior, Rex rabbits

## Abstract

Many studies have shown that stress is associated with gut microbiota. Environmental enrichment (EE) could reduce stress in farm animals; however, limited information is available on the microbial community composition in rabbits raised with or without EE. This study aimed to identify EE influences on the behavior, serum hormonal levels, and cecal microbiota of rabbits. Two hundred Rex rabbits were segregated randomly within four cohorts (*n* = 50); reared for 76 d within standardized enclosures (non-enriched) or within cages containing a willow-stick (WS), rubber-duck (RD), or a can of beans (CB). The rabbits’ ingestive, rest, locomotion, exploratory, grooming, and abnormal behavior were observed. The serum hormone levels for rabbits were measured, and cecal specimens were sequencedfrom the V3–V4 region using 16S rRNA amplicons. Environmental enrichment increased feeding and drinking time, promoted exploratory behavior, and reduced abnormal behavior in rabbits. Insulin-like growth factor 1(IGF-1) levels of the enriched cohorts were elevated in comparison to the control cohort. Serum cortisol level for CB cohort was markedly reduced in comparison to the control cohort (*p* < 0.05), while dopamine levels for CB cohort peaked. Further, we found that EE mainly affected the dominant microbiota. Several families, such as Erysipelotrichaceae, Tannerellaceae, Enterobacteriaceae, Burkholderiaceae, and Prevotellaceae were markedly reduced within the CB cohort. Bacteria such as *Alloprevotella, Bifidobacterium, Enterobacteriaceae, Parabacteroides*, and *Erysipelatoclostridium* were identified as having negative associations with the presence of serum cortisol. EE influenced rabbit behavior and serum hormonal levels, and CB enrichment was the most suitable for rabbits. Further, cecal microbiota composition and diversity were affected by CB enrichment. These findings suggested that CB could be considered for use in rabbit husbandry.

## Introduction

Modern animal husbandry typically involves raising animals under high-density conditions, which can cause environmental stress in animals. Stress can in turn lead to reduced productivity, physical and emotional suffering, and even death (Cheng et al., 2001). Environmental enrichment (EE) improves the environment of captive animals and enhances their physical and psychological well-being by addressing species-specific needs (Vera, 2005). It is important in animal production, because it can relieve environmental pressure, reduce abnormal behavior, and improve animal welfare (Bozicovich et al., 2016). Regarding rabbit production, Princz found that in growing rabbits, gnawing sticks reduced stereotypical behavior, such as cage bar biting or chewing (Princz et al., 2007). Trocino reported that elevated platforms were a useful structural enrichment for improving rabbit behavior (Trocino et al. , 2019). Further, Mohammed & Nasr (2017) found that a wooden stick promoted finalized body-weight, improved several carcass traits, reduced abnormal behavior, while possibly promoting the well-being in rabbits during intensive breeding. However, in previous studies, researchers often used only one type of EE, or solely performed comparative analyses over effects from similar EE resources (*e.g.*, apple-sticks against willow-sticks) (Bozicovich et al., 2016; Princz et al., 2007; Mohammed & Nasr, 2017). Few studies have compared the influences of dissimilar EEs on rabbit behavior. Consequently, identifying the ideal EE resources having peak effectiveness within rabbits can be challenging.

In addition to behavior, hormonal levels are also potential indicators of animal stress. Stress reactions in animals are controlled by their neuroendocrine systems, particularly by the adrenal cortex for the hypothalamic-pituitary-adrenal (HPA) axis and the sympathetic-medullary-adrenal (SMA) axis (Hennessy, 1997). Dopamine (DA) and cortisol are released via these two systems, respectively. These hormones are often associated with stress and are functionally involved in controlling animals’ behavior and metabolic, endocrinal, and immune functions to ensure adequate coping strategies and well-being (Pani, Porcella & Gessa, 2000). Therefore, DA and cortisol levels could reflect the degree of stress in animals.

In recent years, several studies have addressed the role of stress in animal production. Some studies have focused on intestinal—cerebral inter-communication, a pathway known as the brain-gut-microbiota axis (Dinan & Cryan, 2012), and it has been proven that this signalling pathway is bidirectional (Mayer, Savidge & Shulman, 2014). Previous studies have demonstrated that the gut and the central nervous system (CNS) are closely linked and play a role in maintaining gastrointestinal homeostasis, any changes that reduce the beneficial bacteria in the gastrointestinal tract can negatively impact the animals’ neuroendocrine and the immune systems (Taché et al., 2001; Cryan & O’Mahony, 2011). Stress can play a considerable role in dysregulating GIT microbiota constituent levels (Mayer, Savidge & Shulman, 2014). Several evidences suggest that psychological stress may have important effects on the intestinal microbiota of animals and humans (Dinan & Cryan, 2012; Tsilimigras et al., 2018; Partrick et al., 2018). Previous research on animal welfare reported that road transport and rearing-room size affected animals’ cecal microbiota (Ludvigsen, Svihus & Rudi, 2016; Perry et al., 2018). However, few studies have considered the impact of EE on animals’ gastrointestinal microbiota. As EE might reduce stress, we hypothesised that its application could affect animals’ gut microbiota. Due to the association across animals’ general well-being/GIT microbiome harmony, proper knowledge on the effect of EE on gut microbial communities is vital.

Depending upon the biological characteristics of rabbits, we selected three types of EE materials in this study: a willow-stick (typical EE in such animals); a rubber-duck, with multi-sensorial appeal, that could be suspended within the enclosure to satisfy rabbits’ need for exploration, and a can of beans (self-made: we put some mung beans in the tin can) that made a noise when rabbits played with it, which also satisfied rabbits’ curiosity. We investigated which of the three EE materials were most effective in reducing abnormal behavior and reducing stress by measuring the rabbits’ serum hormonal levels and cecal microbiota content. This study represents the first attempt to study the effect of EE on cecum microbiota. Our research findings will offer important guidance to practitioners of rabbit husbandry who seek to enrich rabbits’ environments and improve productivity outcomes.

## Materials and Methods

### Animal, feeding, and housing

The experiment was conducted on July 8–September 24, 2019, at the YuanFa Rex Rabbit Farm in Baiyin, Gansu Province, China (latitude 36°44′N–37°10′N, longitude 104°58′E–05°11′E, and altitude 2,040 m). Two hundred Rex rabbits (2 months old; body weight = 1917 ± 31.45 g) were segregated randomly within four cohorts (*n* = 50): control cohort (CO) and three EE treatment cohorts. From birth to 2 months old, all rabbits were raised under the same conditions, *i.e.*, in a setup of two rabbits/enclosure (60 × 45 × 40 cm; bamboo-floor). They were housed under natural light conditions at a temperature of 12 °C to 24 °C, and relative humidity of 50%–55%. The rabbits were reared for 76 d from the age of 2 months, which represented the fattening stage. They were inspected daily; and allowed access to 4-mm diameter pelleted diet and water *ad libitum*. Diet was prepared according to the dietary nutritional requirements for rabbits given by the Nutritional Research Council. The composition and nutrient levels of the basal diet are shown in Table S1. This investigation was accepted through the Animal Science and Technology, Gansu Agricultural University Animal Care and Use Committee (Approval #2019-2-161). Rabbit care/handling was consistent with the Regulations for the Administration of Affairs Concerning Experimental Animals (The State Science and Technology Commission of P.R. China, 1988). No death or disease in the rabbits was observed during the experimental period.

### Environmental enrichment

**Figure 1:**
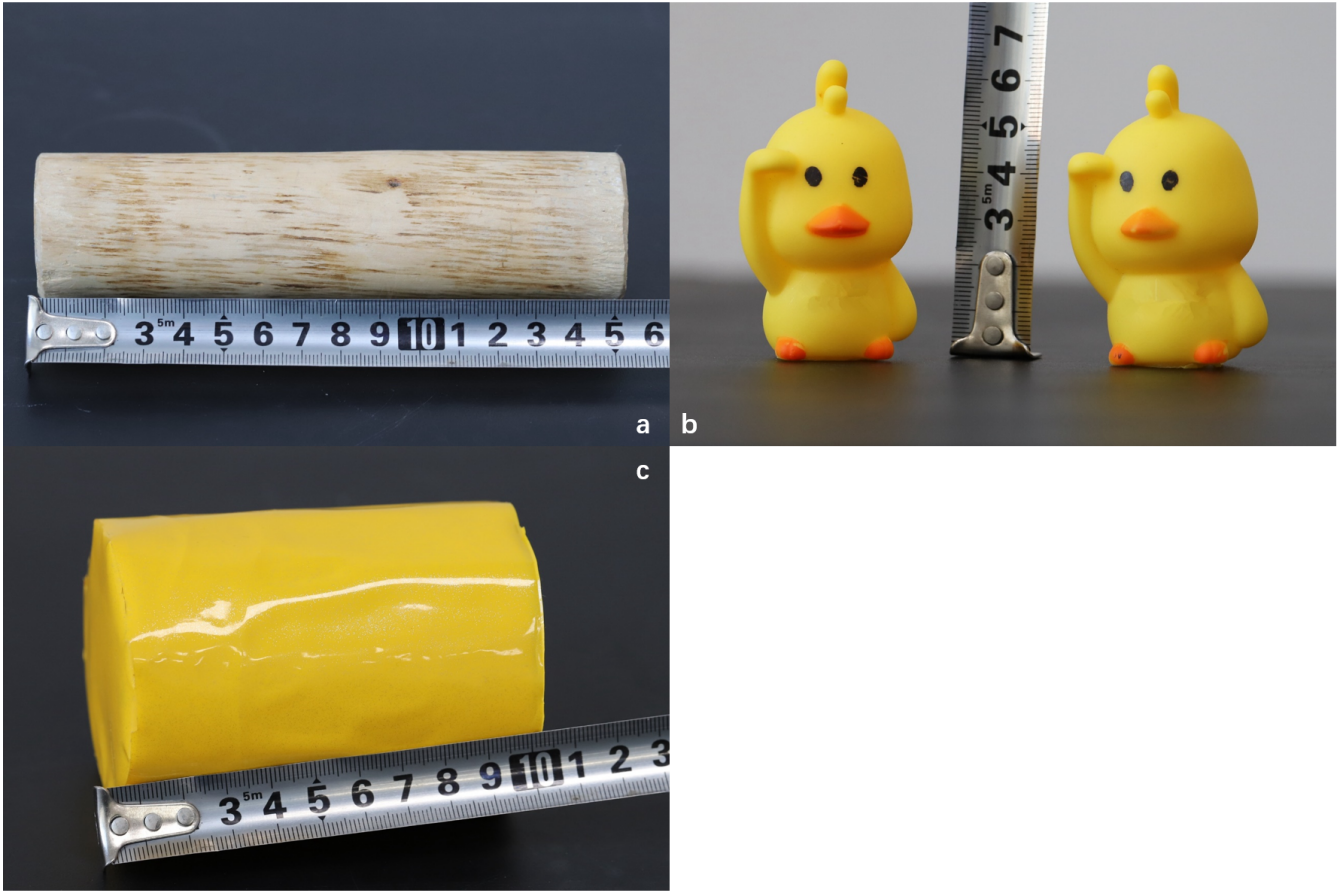
Environmental enrichment materials. (A) Willow stick; (B) rubber duck; (C) can of beans.

Three types of EE material were used in different treatment cohorts: a willow-stick (WS), rubber-duck (RD), and one can of beans (CB)/cage. The RD was hung at approximately 20 cm from the cage bottom, and the WS and CB were placed on the cage floor. The CB could generate sound when rabbits played with it (Fig. 1).

### Behavioral observations

Direct focal observations of rabbits in their home cages were conducted to record different behaviors for 15 consecutive days throughout the experimental period (Abdelfattah et al., 2013). After the preliminary experiment, the behavioral observations began on the 16th day of the experiment. Seventeen observers stood inside the animal enclosure for 10 min before recording their observations to allow the rabbits to acclimatize to their presence. Instantaneous and scan sampling methods were used. To avoid subjective errors, we trained the seventeen observers and assessed their reliability by making them to all record the behavior of one rabbit before the experiment began. The records by the different observers were found to be similar. To minimize the subjective error further, rabbits were randomly assigned to the observers for each observation period. Rabbits were observed twice daily for 40 min each time at noon (12:00) and night (21:40). During the observations, instances of rabbits demonstrating any of the behaviors listed in Table 1 (as defined by Mohammed and Trocino) were recorded (Trocino et al. , 2019; Mohammed & Nasr, 2017).

**Table 1:** Behaviors of Rex rabbits and their definition.

Category	Behavior	Definition
Ingestive behavior	Feeding	Head in feeder
	Drinking	Mouth in contact with drinking nipples
Exploratory behavior	Rearing-up	Hind-leg-based sitting-posture and body in vertical-posture
	Sniffing	Sniffing air / enclosure
	Interaction	Playing or gnawing with cage enrichment material
Abnormal behavior	Circling	Moving in circles
	Abnormal rest posture	Poses other than abdominal-lateral pose, abdominal pose, and lateral pose
	Biting/licking	Wire/feeder gnawing/biting
Locomotion behavior	Walking	Displacing the whole body
	Standing	Standing on the hind legs with front legs on the side for cage
Rest behavior	Rest	Rabbits lying down without any activity (with eyes closed or almost closed)
Grooming behavior	Grooming	Licking/nibbling its fur, forelimbs used for facial cleansing

### Collection of blood specimens and IGF-1, dopamine, and cortisol assays

Rabbit blood specimens were collected on August 29 and 30, 2019. Twenty rabbits per cohort were randomly selected for examining the blood, obtained at 19:00 h before the rabbits received their last daily meal. Auricular arterial blood was obtained via venipuncture, and blood was collected as gently as possible to avoid stress. Blood specimens were placed within serum-separating tubes. To better assist serum separation, the specimens were placed in a water-bath (38 ° C) for 30 min, followed by centrifuging (10 min / 3000 × g) and immediate serum collection/analysis. The serum-hormonal levels were determined using a rabbit IGF-1, DA, and cortisol - stimulating hormone ELISA Kit (HePengBio, Shanghai, China).

### Slaughter, collection of cecal contents, and 16S rRNA gene sequencing

Based on the reduction rule of the “3Rs” experimental animals’ rules, further analysis was conducted depending upon the behavioral and hormonal data. The CB cohort rabbits spent the most time playing with the EE, and their serum hormone levels were distinct in comparison to the CO cohort. Therefore, 16S rRNA genomic-sequencing was employed for characterizing the microbiota constituents within six cecal specimens from the CO and the CB cohorts each. Other rabbits continued to feed until their commercial sale. At the end of the experiment, six rabbits were randomly selected from the two cohorts each for euthanization. The humane endpoint for study was the death of 12 rabbits. They were stunned and exsanguinated via their carotid arteries and jugular veins. The caeca specimens were collected under sterile conditions approximately three cm from the ileocaecal junction and their contents were sampled for microbial DNA extraction. The caecal specimens were snap-frozen within liquid-nitrogen and placed in −80 ° C storage.

Total genomic DNA was collected from each specimen using the CTAB/SDS technique. DNA concentrations/purity were observed using 1% agarose-gels. The extracted DNA was standardized at 1 ng/ µL for polymerase chain reaction (PCR)-template use using bar-coded primers adjacent to the V3–V4 hypervariable-region for bacterial 16S rRNA genome. Primer sequences used were 341F (5′-CCTAYGGGRBGCASCAG-3′)/806R (5′-GGACTACNNGGGTATCTAAT-3′). All PCR runs were performed using 15 µL of Phusion^®^ High-Fidelity PCR Master Mix (New England Biolabs). Sequencing libraries were developed through TruSeq^®^ DNA PCR-Free Sample Preparation Kit (Illumina™, USA) according to the kit protocols, with addition of index-codes. The library-quality was evaluated through Qubit^®^ 2.0 Fluorometer (Thermo Scientific) and Agilent™ Bioanalyser^®^ 2100 platforms. Finally, library sequencing was conducted across the Illumina™ NovaSeq^®^ platform, with the generation of 250 bp paired-end reads. Paired-end reads were merged through FLASH^®^ (V1.2.7), and quality-filtering was performed upon raw-tags with bespoke filtering-conditions for obtaining clean, reliable tags consistent with QIIME (V1.9.1) procedures. All tags were matched to the Silva Database (https://www.arb-silva.de/) through the UCHIME algorithm for identifying the chimeric sequences for exclusion.

### Statistical analysis

For operational taxonomic unit (OTU) production classification, sequence analysis was employed through Uparse v7.0.1001. OTU clustering was performed using UCLUST (97% similarity), and singletons were excluded during downstream evaluations. Representative sequences within individual OTUs were examined for additional annotating. Regarding individual reflecting sequence, the Silva Database was employed along with the Mothur algorithm for annotating taxonomy datasets, and multiple-sequence alignments were conducted using MUSCLE version 3.8.31. Additional evaluations for alpha-/ beta-diversity were conducted depending upon the normalized output datasets. The OTU abundance information was rarefied to the lowest number of reads observed in a single specimen. Beta-diversity was employed for assessing species-complexity-based variations. Beta-diversity analysis was calculated depending upon unweighted UniFrac distances using QIIME version 1.9.1. Metastat analyses were used to evaluate the differences between the two cohorts, while the Benjamini and Hochberg procedures were used for estimating the *q*-value. Cluster analysis was preceded by principal component analysis (PCA), employed for reducing the dimensions for original variables through FactoMine R/ggplot2 packages in R version 2.15.3. Un-weighted pair-cohort technique with arithmetic means (UPGMA) clustering, for hierarchical clustering, was performed for interpreting distance matrix base on mean linkage through QIIME software version 1.9.1.

Other datasets were assessed through SPSS^®^ 20. The alpha diversity index was analyzed using an independent-sample *t*-test. Behavior data obtained repeatedly at multiple sampling times and data from the same cage were considered to be repeated measures and were analysed via repeated-measures analysis of variance (ANOVA). Different behaviors were analysed separately using a generalized linear mixed model that considered EE materials as random effects. Other data were analysed via one-way analysis of variance (ANOVA). Major variations across mean values were determined through Duncan’s test. *p* < 0.05 and *p* < 0.01 were deemed to confer statistical significance, and dataset outcomes were presented as means ± standard error of means (SEM).

## Results

### Behavior observations

**Figure 2:**
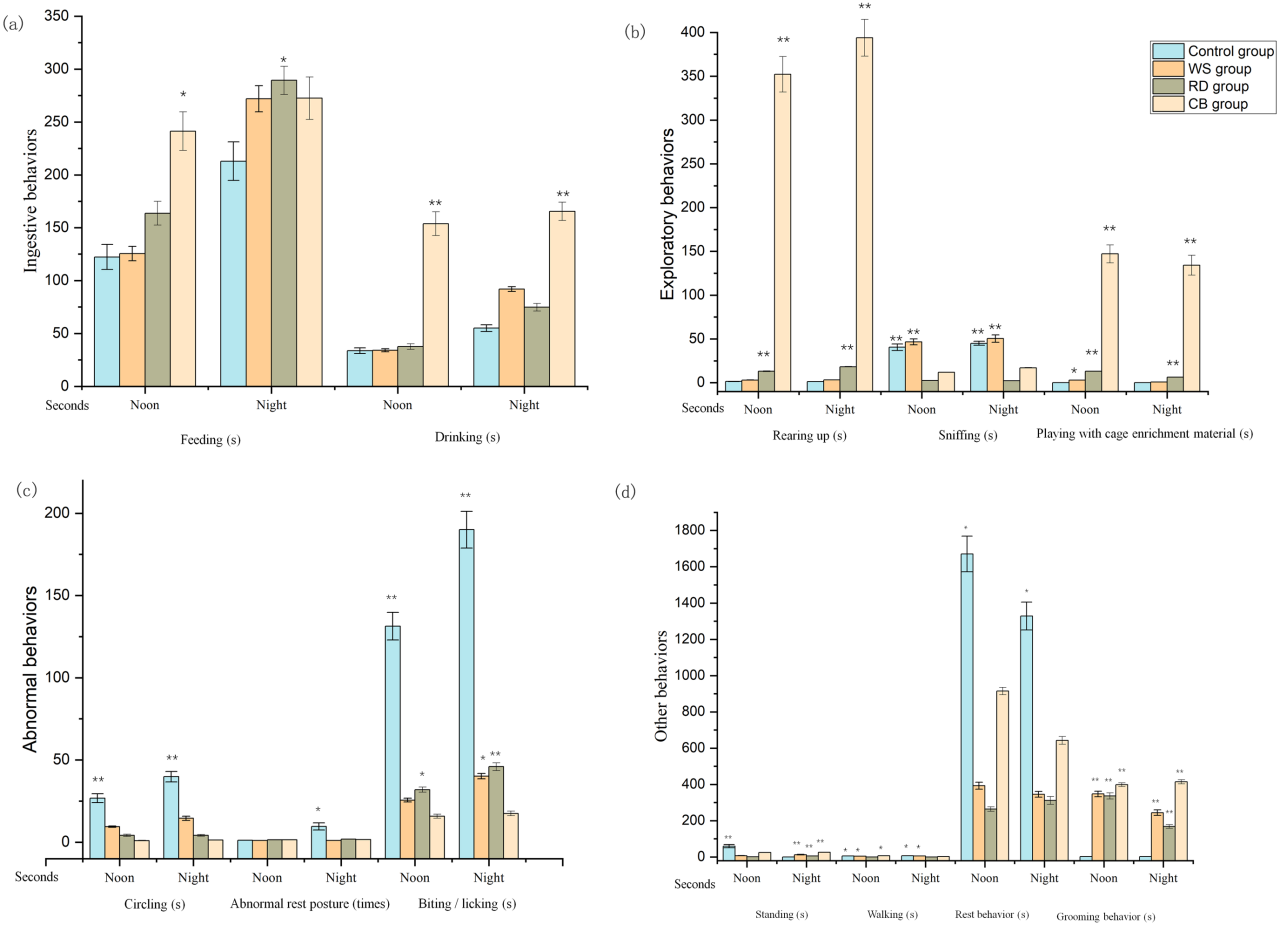
Effect of different environmental enrichment (EE) materials on rabbits’ behavior (during the observation periods). (A) Ingestive behavior; (B) Exploratory behavior; (C) Abnormal behavior; (D) Standing, walking, resting, and grooming behavior. CO, control; WS, willow stick; RD, rubber duck; CB, Can of beans. (s) Measured as duration; (times) Counted as instances of behavior. ^∗^ *p* < 0.05, ^∗∗^ *p* < 0.01; values not reported where *p* > 0.05.

As shown in Fig. 2, the feeding time for enriched cohorts was more elevated than that for the CO cohort at noon and night. The feeding (noon) and drinking (noon and night) times of rabbits within CB cohort were markedly longer in comparison to the CO-cohort rabbits (*p* < 0.05 and *p* < 0.01 respectively). Regarding exploratory behavior, rearing-up behavior occurred more frequently in both the RD and CB cohorts than within the CO and WS cohorts at noon and night (*p* < 0.01). The sniffing time for RD- and CB- cohort rabbits was less than for those in the CO and WS cohorts (*p* < 0.01). Rabbits played with the CB longer than with the other forms of enrichment and spent the least amount of time playing with the WS. The circling time for the enriched cohort was significantly less in comparison to the CO cohort (*p* < 0.01). At night, fewer abnormal rest postures were observed within the EE cohorts than within the CO cohort (*p* < 0.05). Further, less incidences of biting were observed within the EE cohorts than within the CO cohort, and the CB cohort demonstrated the least number of biting instances, which was markedly less than that within the CO cohort (*p* < 0.01). Interestingly, the EE cohorts demonstrated significantly less standing behavior than the CO cohort at noon, while the opposite trend was observed at night (*p* < 0.01). The RD cohort spent the least amount of time walking (*p* < 0.05), and the walking time for the CB cohort was also significantly less in than for the CO cohort (*p* < 0.05). Rabbits within the enriched cohorts spent less time resting than those within CO cohort at noon and night (*p* < 0.05). Grooming behavior within the enriched cohorts was markedly more prevalent in comparison to within the CO cohort (*p* < 0.01).

### IGF-1, dopamine, and cortisol levels

As shown in Table 2, the IGF-1 levels of rabbits within the enriched cages were markedly elevated in comparison to within the CO cohort (*p* < 0.05). The DA levels for the CB cohort were markedly elevated in comparison to other cohorts (*p* < 0.05). Serum cortisol levels for the CO cohort were markedly elevated in comparison to the WS/CB cohorts.

### Cecum microbiota

We acquired 1,156,501 high-quality paired-end sequences, with mean read length of 411 bp/specimen. Depending upon a 97% species-similarity-threshold, 1,105 OTUs were found from the specimens. Further, 13 phyla, 17 classes, 22 orders, 39 families, 73 genera, and 70 species were identified.

**Figure 3:**
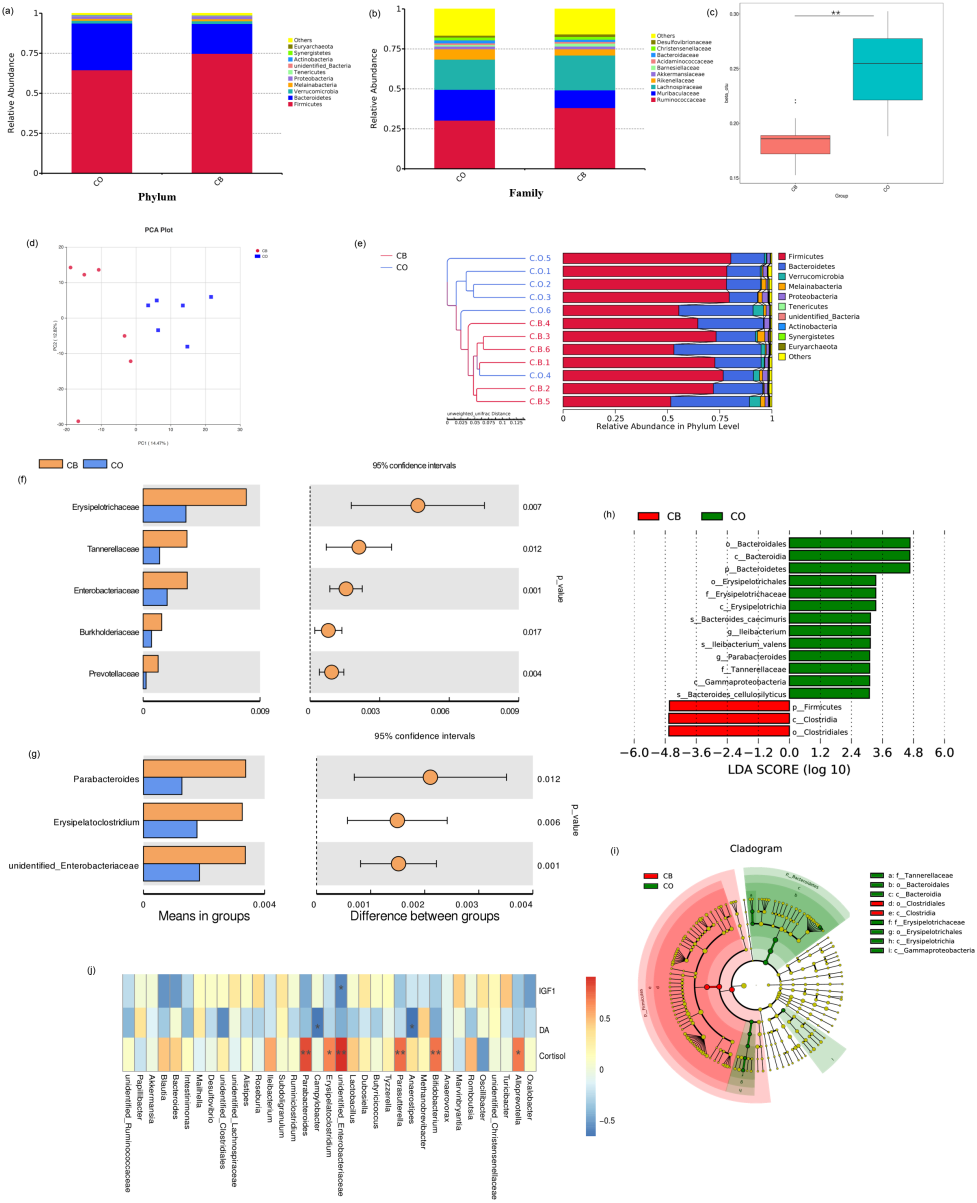
Comparison of cecal microbiota of rabbits in the CO and CB groups. Mean relative abundance for the 10 best-ranking phyla (A)/top10 families (B) within the CB-and CO -cohort rabbits; (C) Box-plot of cecal microbial *β*-populations (Wilcoxon rank-sum test, *p* < *0*.01); (D) PCA for rabbit cecal specimens depending upon unweighted UniFrac distances; (E) Similarity-cluster analyses of rabbit cecal specimens through UPGMA. (F) Bacterial taxa differences at the family level; (G) bacterial taxa differences at the genus level. Bacterial taxa having mean relative presence > 0.1% within a minimum of one cohort were encompassed; (h) Linear discriminant analysis (LDA) diagram of taxonomic differences between CO- and CB- cohort rabbits depending on LEfSe analysis. Species having major variations regarding presence with an LDA score > 3.0.Histogram bar reflects LDA scoring; (i) Cladogram demonstrates micro-organism-based populations that exhibited major variations across both cohorts. Red and green within phylogenetic-tree reflect micro-organism-based populations having pivotal parts within CB and CO cohorts respectively; (J) Heat map of Spearman correlation analysis results between bacterial genera and serum hormonal levels of rabbits. * *p* < 0.05; ^∗∗^ *p* < 0.01, CO, control; CB, Can of beans.

Relative presence for top-ranking ten phyla and microbial families present within CO- and CB- cohort rabbits are shown in Fig. 3. Firmicutes / Bacteroidetes predominated as phyla within both CO and CB cohorts. Firmicutes accounted for 74.8% for families within the CO cohort and 64.5% within the CB cohort (Fig. 3A). Ruminococcaceae and Lachnospiraceae were the most prevalent bacteria families within the CO and CB cohorts (Fig. 3B).

**Table 2:** IGF-1, dopamine, and cortisol levels in rabbits reared in enriched and conventional cages.

Trait	CO cohort (*n* = 20)	WS cohort (*n* = 20)	RD cohort (*n* = 20)	CB cohort (*n* = 20)	*p*-value
IGF-1 ng/mL	85.57 ± 29.26^b^	119.47 ± 23.07^a^	116.59 ± 17.07^a^	105.21 ± 7.04^a^	0.023
Dopamine nmol/L	31.22 ± 5.44^b^	30.93 ± 7.13^b^	33.41 ± 7.02^b^	40.58 ± 14.22^a^	0.039
Cortisol ng/mL	108.47 ± 34.46^a^	80.42 ± 10.72^b^	84.90 ± 14.19^b^	76.58 ± 10.20^b^	0.036

**Notes.**

a, b signify values that differ significantly (*p* <0.05).

CO control WS willow stick RD rubber duck CB can of beans

The observed_species, PD_whole_tree, and Ace indices were markedly elevated within the CO cohort than within the CB cohort. Alternative alpha diversity indices for cecal microbiota in rabbits raised with and without EE did not differ significantly (Table 3).

**Table 3:** Cecal bacterial alpha diversity in the CO- and CB- cohort rabbits.

	CO cohort (*n* = 6)	CB cohort (*n* = 6)	*p*-value
Observed_species	880.0 ± 14.95	787.17 ± 27.76	0.015
Goods_coverage	0.9981 ± 0.00013	0.99 ± 0.00017	0.554
Shannon	6.70 ± 0.20	6.99 ± 0.10	0.949
PD_whole_tree	62.91 ± 0.86	56.63 ± 1.65	0.007
Simpson	0.96 ± 0.01	0.98 ± 0.001	0.217
Chao1	926.60 ± 16.42	846.05 ± 32.87	0.053
Ace	925.64 ± 15.00	841.66 ± 30.85	0.034

**Notes.**

CO control CB Can of beans

A major variation was found within beta diversity for the OTU structures between the CO and CB cohorts (Wilcoxon rank-sum test: *p* < 0.01). Beta diversity was markedly reduced within the CB cohort than within CO cohort (Wilcoxon rank-sum test: *p* < 0.01; Fig. 3C, Table S2), indicating that CB enrichment caused low variance across cecal microbiota constituent make-up within rabbits. PCA trajectory plot also revealed distinctions between the microbiota communities within the CO and CB cohorts (Fig. 3D). Similarity-cluster analyses through UPGMA demonstrated adequate corroboration with the PCA analyses (Fig. 3E), suggesting that cecal micro-flora within rabbits changes with the progressive change in the environment caused by EE materials.

We performed a Linear discriminant analysis Effect Size (LEfSe) analysis to reveal differences within significance ranking of abundant bacterial taxa within the CB- and CO- cohort specimens (Fig. 3H). Fig. 3I demonstrates nine valuable microbial taxa. Within CB cohort, Clostridia and the order Clostridiales were important biomarkers. Biomarkers within the CO cohort included Bacteroidia (c), Erysipelotrichia (h), and Gammaproteobacteria (i). Variations were assessed within main bacterial taxa (mean relative presence >0.01% across both cohorts) for the CO/CB cohort rabbits using the *t*-test. For the two cohorts, five families (Fig. 3F) and three genera (Fig. 3G) showed significant differences between the CO and CB cohort rabbits. Similar microbial taxa were found through metastat analysis (Fig. S1) between the CO and CB cohort rabbits.

Spearman’s correlation was conducted for microbial genera/serum hormone levels (IGF-1, DA, and cortisol) in rabbits. As shown in Fig. 3J, a negative correlation was observed between *Enterobacteriaceae* and IGF-1 (*p* < 0.05). DA was positively correlated to *Anaerostipes* and *Campylobacter* (*p* < 0.05). *Bifidobacterium, Parasutterella, Parabacteroides*, and *Enterobacteriaceae* showed a strong positive correlation with cortisol levels (*p* < 0.01). Additionally, *Erysipelatoclostridium* and *Alloprevotella* showed a positive correlation with cortisol levels (*p* < 0.05).

FAPROTAX was employed in microbial functional assessment. Ecological roles for bacteria/archaea within gut specimens were classified through FAPROTAX. Some gut/nitrate functions for bacteria showed significant differences between the two cohorts (Fig. S2).

## Discussion

### Effects of environmental enrichment on behavior

Across all EE cohorts, CB was favoured by the rabbits, and they played the least with WS (Noon: 153.83 s *vs*. 2.97 s; Night: 134.17 s *vs*. 0.75 s for CB *vs*. WS). This may be attributable to the sound produced by CB when the rabbits played with it, which stimulated the animals’ desire for exploration. As expected, rabbits that spent more time playing with EE rested less during the daytime. The time that rabbits spent in comfortable resting (*i.e.*, in a stretched position) within enriched cages was less than within the CO cohort. The expanded behavioral opportunities offered by the various forms of enrichment seemed to reduce the time rabbits spent lying down, as reported previously by Luzi et al. (2003). While feeding time at night and drinking time at noon and night were all longer among the EE-cohort rabbits than among the CO- cohort rabbits, the feeding and drinking time for the CB cohort was the longest. This indicated that EE could increase rabbits’ appetites and may promote weight gain in animals. The results of previous studies conducted by Luzi et al. (2003), Princz et al. (2005) and Mohammed & Nasr (2017) support this hypothesis and suggest that EE promotes weight gain in rabbits. In our trial, EE also had a positive effect on other behaviors. Exploratory behaviors (rearing-up and playing) were more common within the EE cohorts in comparison to within CO cohort. However, sniffing time was reduced within RD and CB cohorts, and we speculate that the increased time and attention devoted to playing with the EE shortened the rabbits’ sniffing time. At night, abnormal resting postures were increasingly observed within the CO-cohort rabbits than in the EE-cohort rabbits. The RD- cohort rabbits spent the least amount of time standing and walking but there was no obvious trend of standing and walking in other cohorts. This may reflect the fact that the RD that hung from the cage prevented the rabbits from standing and walking to some extent. Circling and biting are usually considered stereotypical behaviors of captive rabbits (Trocino et al. , 2019; Seidel, Beaton & Teague, 1979), and the decreased circling and biting behaviors among the EE-cohort rabbits may suggest that the EE re-directed the rabbits’ attention. These findings support those of previous studies, which showed that EE reduced abnormal behavior in growing rabbits (Mohammed & Nasr, 2017; Abdelfattah et al., 2013). We believe this is because EE relieved boredom and satisfied ethological needs. The increase in grooming behavior within present study diverges from who provided a wooden enrichment structure for rabbits and found that grooming behavior was reduced (Trocino et al., 2019). This might be attributable to the different types of EE and experimental methods used in the two studies.

### Effect of environmental enrichment on serum hormone levels

Insulin-like growth factor 1 regulates cell proliferation and plays an important role in cell differentiation, proliferation, and individual growth development (Holzenberger et al., 2003). The high serum IGF-1 levels observed in EE-cohort rabbits indicate that EE could affect the production of serum IGF-1 and rabbit growth. Cortisol and DA are hormones associated with stress (Cheng et al., 2001; Barik et al., 2013). Animals’ stress reactions are controlled by their neuroendocrine systems. Cortisol is released from the adrenal cortex of the HPA axis, and DA is released from peripheral systems, including the medulliadrenal SMA axis (Hennessy, 1997). Both axes are important common pathways in controlling animals’ ability to cope with their environments and their responses to stressors (Negro et al., 2000; Ehlert, Gaab & Heinrichs, 2001). DA and cortisol are functionally involved in controlling an organism’s behavioral tendencies and metabolic, endocrine, and immune functions, and work to ensure adequate coping strategies and individual well-being (Cheng et al., 2001; Pani, Porcella & Gessa, 2000). Animals in stressful environments present high cortisol levels, Staay et al. (2010) and De Vry et al. (2012) found that the blood cortisol levels of pregnant sows raised in restrictive-breeding environments were markedly elevated in comparison to raised in cohort-breeding environments, Pavičić et al. (2003) and Leme et al. (2012) reported that transportation is a stressor that impacts plasmatic cortisol levels in lambs and pigs. In this study, the serum cortisol concentrations within enriched cohorts were reduced in comparison to within CO cohort (Table 3, *p* < 0.05), suggesting that EE decreased stress. Dopamine is also associated with stress, along with various comorbidities including insomnia, chronic pain, and depression (Finan & Smith, 2013). A moderate increase in dopamine within a certain range is beneficial for animals, but overly high DA is linked to increased aggressive behavior, cannibalism, and elevated mortality (Cheng et al., 2001; Craig & Muir, 1996). In our trial, aggressive behavior was not observed in EE-cohort rabbits. Moreover, CB-cohort rabbits, which had the highest DA levels, spent more time playing with EE, demonstrated less abnormal behavior (biting wire or abnormal rest postures), and displayed the most positive behavior among cohorts. This suggested that rabbits experienced less stress within the CB-enriched cage, and consequently, their DA and cortisol levels were the highest and lowest, respectively. Under the experimental conditions of this study, CB was found to be the most suitable EE for the rabbits.

### Effect of environmental enrichment on cecal microbiota

Not many investigations have analysed associations across environmental enriched cages/gut microbiota within animals. To our knowledge, this is the first investigation to report influences of EE upon cecal microbiota within rabbits. The relationship between the mental health and gut microbial is more and more be accounted of researcher. The gut microbiota interacts with the host via neuroimmune, neuroendocrine and neural pathways. Gut -brain communication has been explored in many animal models. The brain-gut-microbiota axis and preclinical evidence suggests that the microbiota can recruit this bidirectional communication system to modulate brain development, function and behavior (Cryan & Dinan, 2012). A study in human have been provided that gut metabolite has effect the mental heath (Valles-Colomer et al., 2019). Some evidences indicated that gut microbiota may play a causal role in the development of features of depression (Kelly et al., 2016). Our data demonstrated that phyla such as Firmicutes and Bacteroidetes dominate the rabbit cecal ecosystem, representing more than 90% for the entire microbial composition of both CO- and CB- cohort rabbits. This was in accordance with the previous studies that have characterized the caecal microbiota in rabbits and reported that Firmicutes and Bacteroidetes are the predominant phyla in the New Zealand White and Rex rabbit cecal microbial communities (Chen et al., 2019; Zou et al., 2016). Conversely, other investigations mapped cecal microbiota within meat rabbits and showed elevated relative presence of Proteobacteria and Verrucomicrobia phyla (Kylie, Weese & Turner, 2018). These discrepancies among previous studies could be attributable to technical issues or biological reasons. Within the present study, the CB-cohort rabbits showed reduced relative abundances of Firmicutes and increased abundances of Bacteroidetes relative to those within other cohorts. Although no direct comparisons can be made between this study and others, elevated Firmicutes populations and reduced Bacteroidetes populations were observed among rabbits raised in more congenial environments (Velasco-Galilea et al., 2020). This may indicate that the addition of EE provided rabbits with healthier environments.

Regarding the alpha diversity assessment, the observed_species, Ace, and PD_whole_tree indices revealed significant differences between the CO and CB cohorts. Cecal specimens collected from the CO-cohort rabbits were more diverse in comparison to those from the CB-cohort rabbits. However, the Shannon diversity-index in the CB cohort was more elevated than in the CO cohort (not significant). Further, EE may favour the presence of only certain gut microbiota. Although no direct comparisons can be made between the present and previous studies, we found that our results were inconsistent with the results of other studies, which indicated that positive environments were associated with elevated alpha diversity in chickens and horses (Ludvigsen, Svihus & Rudi, 2016; Adhikari et al., 2020). Further research is needed to explain these differences.

Additionally, our study showed that EE affected several intestinal bacterial families within rabbits under identical diets/environmental conditions (except for the presence or absence of EE), namely Erysipelotrichaceae, Tannerellaceae, Enterobacteriaceae, Burkholderiaceae, and Prevotellaceae, all of which markedly increased within the CO cohort than within the CB cohort (Fig. 3). Within human and rodent models, Erysipelotrichaceae was linked to high-fat diets (Martinez et al., 2009; Fleissner et al., 2010) and several diseases, including inflammation-linked GIT conditions/metabolic disorders (Kaakoush, 2015; Bermingham et al., 2017). Similarly, Tannerellaceae and Enterobacteriaceae are often associated with gastrointestinal diseases (Winsen et al., 2002; Lopetuso et al., 2018). In this study, however, diarrhea and gastrointestinal diseases were not observed within the CO-cohort rabbits. Enterobacteriaceae, Burkholderiaceae, and Prevotellaceae were members for the Proteobacteria phylum. Increased Proteobacteria populations are associated with anxiety and further impact stress-disturbed gut microbiota compositions (Hyo-Min et al., 2018). These results may be attributable to reduced stress and anxiety under conditions with EE, and changes in rabbit well-being may further affect Proteobacteria populations. In the present study, considerable links across multiple genera and host serum cortisol levels were identified within rabbits. Presence of *Alloprevotella, Bifidobacterium*, Enterobacteriaceae, *Parabacteroides,* and *Erysipelatoclostridium* was strongly positively associated with host serum cortisol levels. The identical profile, recognized through elevated cortisol discharge, was found in horses exposed to stressors and in rhesus monkeys prenatally exposed to an acoustic stressor (Bailey, Lubach & Coe, 2004; Mach et al., 2017). Although still hypothetical, the mechanisms for cortisol discharge in driving gut microbiota constitutional dysregulations could be associated with stress-influenced shifts within the intestinal physiology that shifts bacterial colonies (*e.g.*, alteration of gut permeability and barrier function or bile acid concentrations) to interbacterial signalling, growth, and virulence (Cryan & Dinan, 2012).

**Figure 4:**
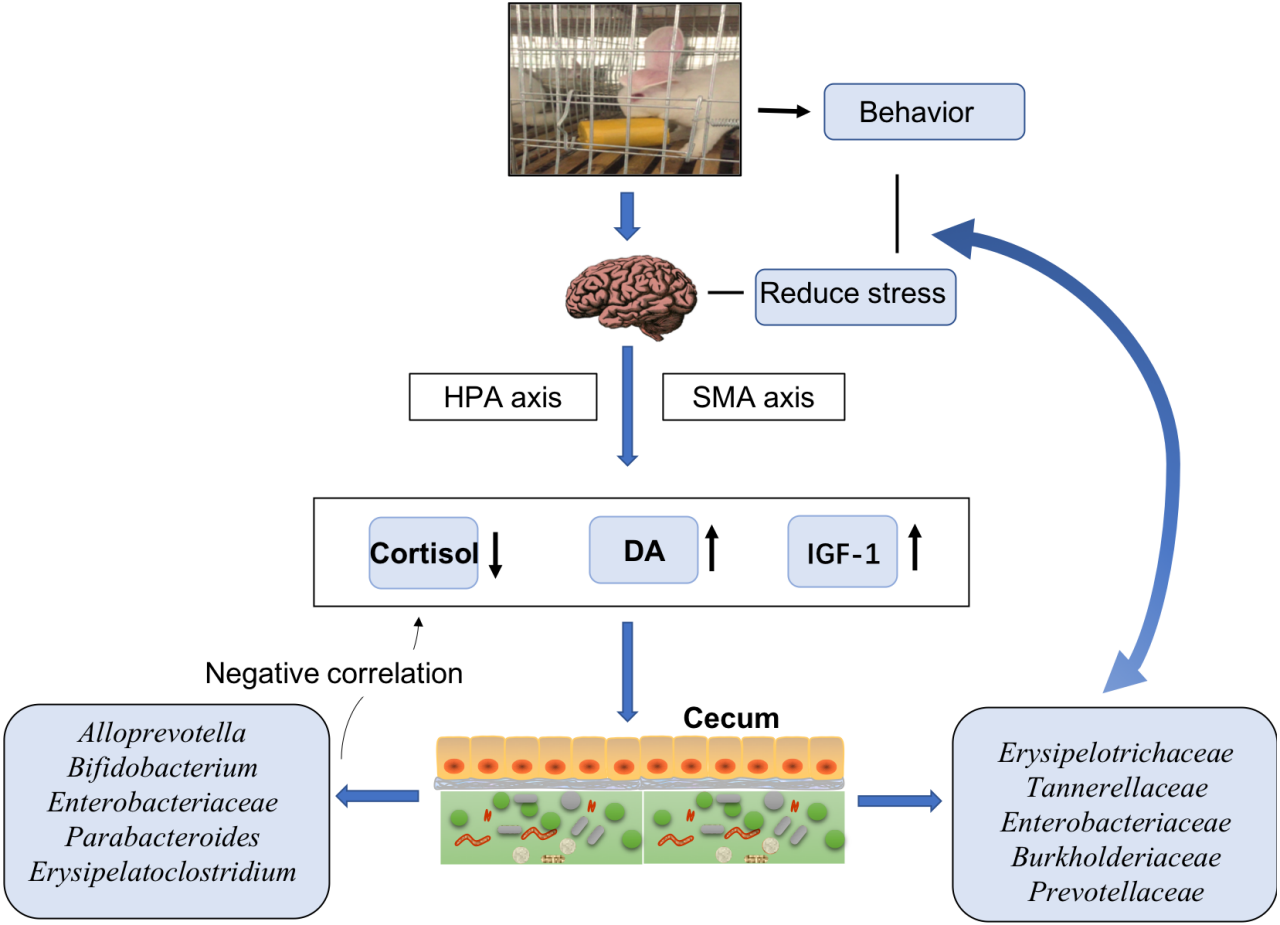
Model of cecal microbiota variations and their effects on host physiology under enriched environments.

In general, our findings suggested that EE changed the rabbits’ behavior, stimulated their HPA and SMA axes, decreased the release of cortisol, and increased the release of DA and IGF-1. Further effects possibly attributable to EE included alterations in the cecal microbiota with decreased abundances of Erysipelotrichaceae, Tannerellaceae, Enterobacteriaceae, Burkholderiaceae, and Prevotellaceae. These bacteria communicate with the brain to reduce stress and decrease the anxiety in animals. Moreover, bacteria such as *Alloprevotella, Bifidobacterium,* Enterobacteriaceae, *Parabacteroides*, and *Erysipelatoclostridium* had negative links with serum cortisol levels. Most of such bacterial taxa displayed markedly different relative abundances between the CO and CB cohorts. These findings suggested a possible effect of EE on stress responses in hosts (Fig. 4).

## Conclusion

Rabbits’ behavior and serum hormonal levels were influenced by three different types of EE, among which, CB was found to be the most suitable. Furthermore, our results confirmed that rabbits raised in the CB enriched cages had more microbiota characteristic of healthy animals compared to rabbits in conventional cages. Psychological stress can alter the composition of animals’ intestinal microbiota, and the brain-gut-microbiota interactions involved in regulating the effects of stress on intestinal functions are now better understood. As animal husbandry practices often involve animals under stress, EE could represent a useful stress-reduction method. Although an extensive study is required to further explore these relationships, we suggest that the characteristics of enclosures should be given greater consideration in rabbit husbandry.

## Supplemental Information

10.7717/peerj.13068/supp-1 Supplemental Information 1 Supplemental tables

10.7717/peerj.13068/supp-2 Supplemental Information 2 The metastat between the two groups

10.7717/peerj.13068/supp-3 Supplemental Information 3 The FAPROTX analysis of the CO and CB cohorts

10.7717/peerj.13068/supp-4 Supplemental Information 4 Raw data of CB1 sample sequenced by 16S rRNA gene V3-V4 region Illumina MiSeq: Part 1

10.7717/peerj.13068/supp-5 Supplemental Information 5 Raw data of CB1 sample sequenced by 16S rRNA gene V3-V4 region Illumina MiSeq: Part 2

10.7717/peerj.13068/supp-6 Supplemental Information 6 Raw data of CB2 sample sequenced by 16S rRNA gene V3-V4 region Illumina MiSeq: Part 1

10.7717/peerj.13068/supp-7 Supplemental Information 7 Raw data of CB2 sample sequenced by 16S rRNA gene V3-V4 region Illumina MiSeq: Part 2

10.7717/peerj.13068/supp-8 Supplemental Information 8 Raw data of CB3 sample sequenced by 16S rRNA gene V3-V4 region Illumina MiSeq: Part 1

10.7717/peerj.13068/supp-9 Supplemental Information 9 Raw data of CB3 sample sequenced by 16S rRNA gene V3-V4 region Illumina MiSeq: Part 2

10.7717/peerj.13068/supp-10 Supplemental Information 10 Raw data of CB4 sample sequenced by 16S rRNA gene V3-V4 region Illumina MiSeq: Part 1

10.7717/peerj.13068/supp-11 Supplemental Information 11 Raw data of CB4 sample sequenced by 16S rRNA gene V3-V4 region Illumina MiSeq: Part 2

10.7717/peerj.13068/supp-12 Supplemental Information 12 Raw data of CB5 sample sequenced by 16S rRNA gene V3-V4 region Illumina MiSeq: Part 1

10.7717/peerj.13068/supp-13 Supplemental Information 13 Raw data of CB5 sample sequenced by 16S rRNA gene V3-V4 region Illumina MiSeq: Part 2

10.7717/peerj.13068/supp-14 Supplemental Information 14 Raw data of CB6 sample sequenced by 16S rRNA gene V3-V4 region Illumina MiSeq: Part 1

10.7717/peerj.13068/supp-15 Supplemental Information 15 Raw data of CB6 sample sequenced by 16S rRNA gene V3-V4 region Illumina MiSeq: Part 2

10.7717/peerj.13068/supp-16 Supplemental Information 16 Raw data of CO1 sample sequenced by 16S rRNA gene V3-V4 region Illumina MiSeq: Part 1

10.7717/peerj.13068/supp-17 Supplemental Information 17 Raw data of CO1 sample sequenced by 16S rRNA gene V3-V4 region Illumina MiSeq: Part 2

10.7717/peerj.13068/supp-18 Supplemental Information 18 Raw data of CO2 sample sequenced by 16S rRNA gene V3-V4 region Illumina MiSeq: Part 1

10.7717/peerj.13068/supp-19 Supplemental Information 19 The microbiota sequencing date of CO2Raw data of CO2 sample sequenced by 16S rRNA gene V3-V4 region Illumina MiSeq: Part 2

10.7717/peerj.13068/supp-20 Supplemental Information 20 Raw data of CO3 sample sequenced by 16S rRNA gene V3-V4 region Illumina MiSeq: Part 1

10.7717/peerj.13068/supp-21 Supplemental Information 21 Raw data of CO3 sample sequenced by 16S rRNA gene V3-V4 region Illumina MiSeq: Part 2

10.7717/peerj.13068/supp-22 Supplemental Information 22 Raw data of CO4 sample sequenced by 16S rRNA gene V3-V4 region Illumina MiSeq: Part 1

10.7717/peerj.13068/supp-23 Supplemental Information 23 Raw data of CO4 sample sequenced by 16S rRNA gene V3-V4 region Illumina MiSeq: Part 2

10.7717/peerj.13068/supp-24 Supplemental Information 24 Raw data of CO5 sample sequenced by 16S rRNA gene V3-V4 region Illumina MiSeq: Part 1

10.7717/peerj.13068/supp-25 Supplemental Information 25 Raw data of CO5 sample sequenced by 16S rRNA gene V3-V4 region Illumina MiSeq: Part 2

10.7717/peerj.13068/supp-26 Supplemental Information 26 Raw data of CO6 sample sequenced by 16S rRNA gene V3-V4 region Illumina MiSeq: Part 1

10.7717/peerj.13068/supp-27 Supplemental Information 27 Raw data of CO6 sample sequenced by 16S rRNA gene V3-V4 region Illumina MiSeq: Part 2

10.7717/peerj.13068/supp-28 Supplemental Information 28 The *T*-test of microbes between CB and CO group in phylum level

10.7717/peerj.13068/supp-29 Supplemental Information 29 The *T*-test of microbes between CB and CO group in class level

10.7717/peerj.13068/supp-30 Supplemental Information 30 The *T*-test of microbes between CB and CO group in family level

10.7717/peerj.13068/supp-31 Supplemental Information 31 The *T*-test of microbes between CB and CO group in genus level

10.7717/peerj.13068/supp-32 Supplemental Information 32 The *T*-test of microbes between CB and CO group in order level

10.7717/peerj.13068/supp-33 Supplemental Information 33 The *T*-test of microbes between CB and CO group in species level

10.7717/peerj.13068/supp-34 Supplemental Information 34 Protocol registration statement

10.7717/peerj.13068/supp-35 Supplemental Information 35 Author checklist

## Additional Information and Declarations

### Data Availability

The following information was supplied regarding data availability: The raw measurements are available in the Supplementary Files.
