# Supplementary material for: Environmental enrichment changes rabbits’ behavior, serum hormone level and further affects cecal microbiota
Source: PeerJ. 2022 Mar 9;10:e13068. doi: 10.7717/peerj.13068 (PMC8917805; doi:10.7717/peerj.13068)
Supplement: Supplemental Information 1 [file peerj-10-13068-s001.docx]

Table S1 Composition and nutrient levels of the basal diet (air-dry basis)

| **Ingredient** | **Level (%)** |
| --- | --- |
| Alfalfa meal | 62.80 |
| Corn | 15.99 |
| Wheat bran | 6.11 |
| Soybean meal | 6.51 |
| Greaves | 4.08 |
| Premix | 4.00 |
| NaHCO_3_ | 0.09 |
| Allicin | 0.10 |
| NaCl | 0.32 |
| **Nutrients** | **Concentration** |
| DE/ (MJ/kg) | 10.35 |
| CP（%） | 17.52 |
| CF（%） | 15.56 |
| Ca（%） | 0.83 |
| P（%） | 0.41 |

DE: digestible energy; CP: crude protein; CF: crude fiber; Ca: calcium; P: phosphorus.

Premix provided the following per kg of the diet: vitamin A – 8,000IU; vitamin B1 - 1.8mg; vitamin B2 - 6mg; vitamin B6 - 0.3mg; vitamin D - 800IU; vitamin E 50 mg; Cu 50mg; Fe 100mg; Zn 50mg; Mn 30mg; Mg 150mg; Se 0.1mg.

Table S2. The statistics of beta diversity by Adonis

| Group | Df | Sums of Sqs | Mean Sqs | F.Model | R2 | Pr (>F) |
| --- | --- | --- | --- | --- | --- | --- |
| CO-CB | 1(10) | 0.08216 | 0.082159 | 2.2931 | 0.18653 | 0.006 |

Base on the binary jaccard.
