# Supplementary figures and images for: Environmental enrichment changes rabbits’ behavior, serum hormone level and further affects cecal microbiota
Source: PeerJ. 2022 Mar 9;10:e13068. doi: 10.7717/peerj.13068 (PMC8917805; doi:10.7717/peerj.13068)

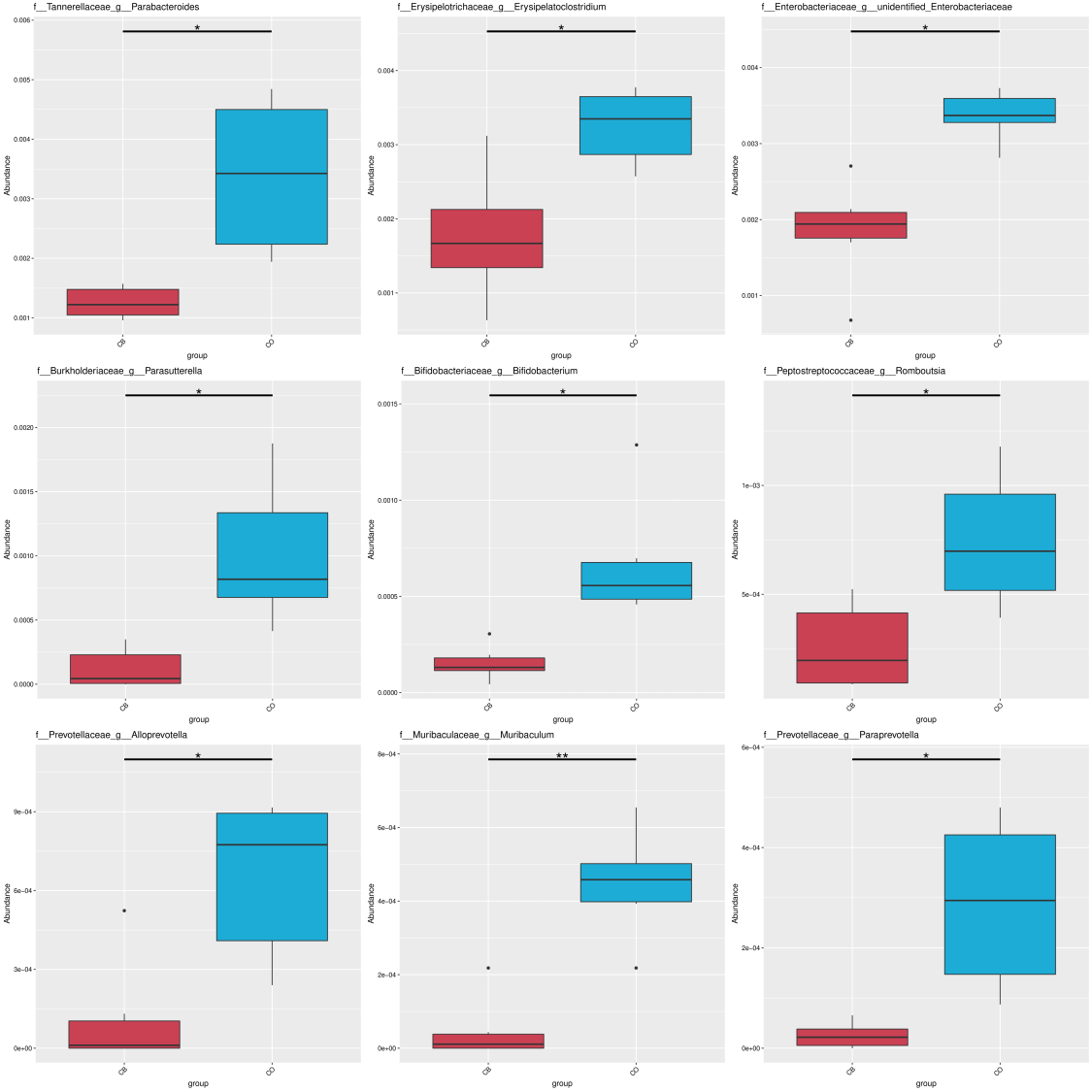

Supplement: Supplemental Information 2 [file peerj-10-13068-s002.png]

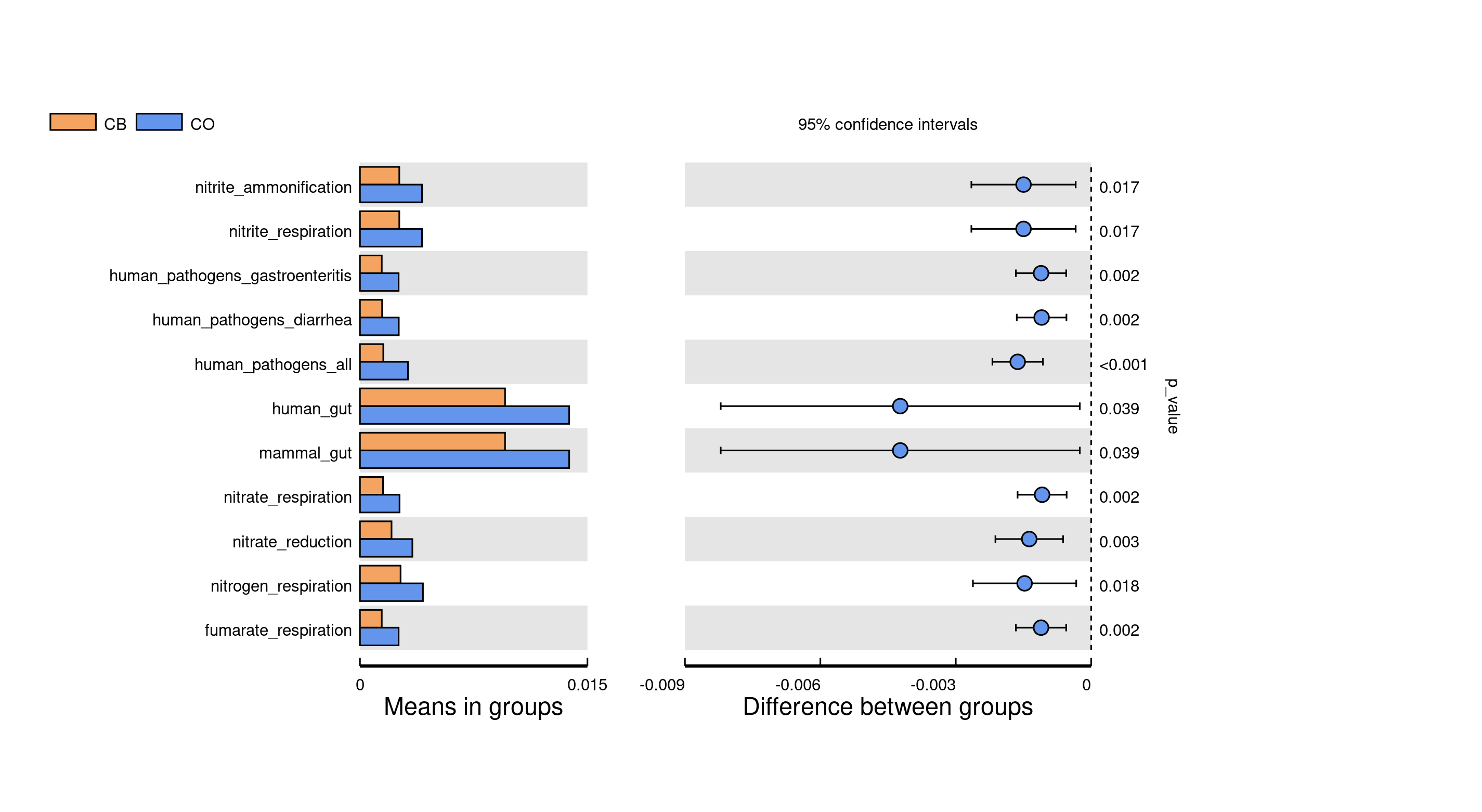

Supplement: Supplemental Information 3 [file peerj-10-13068-s003.png]
