# Supplementary material for: Environmental enrichment changes rabbits’ behavior, serum hormone level and further affects cecal microbiota
Source: PeerJ. 2022 Mar 9;10:e13068. doi: 10.7717/peerj.13068 (PMC8917805; doi:10.7717/peerj.13068)
Supplement: Supplemental Information 34 [file peerj-10-13068-s034.docx]

**Statement**

Before the study, we made the protocol about the research question, key design features, analysis plan, and registered in the College Animal Science and Technology, Gansu Agricultural University.

Corresponding author:

Shuangbao Gun

E-mail: gunsb@gsau.edu.cn
